# Estimated Percentages and Characteristics of Men Who Have Sex with Men and Use Injection Drugs — United States, 1999–2011

**Published:** 2013-09-20

**Authors:** Norma Harris, Christopher Johnson, Catlainn Sionean, Wade Ivy, Sonia Singh, Stanley Wei, Yuko Mizuno, Amy Lansky

**Affiliations:** Div of HIV/AIDS Prevention, National Center for HIV, Viral Hepatitis, STD, and TB Prevention, CDC

Male-to-male sex and illicit injection drug use are important transmission routes for human immunodeficiency virus (HIV) infection. Of all new HIV infections in 2010, 80% were among men, of which 78% were among men who have sex with men (MSM), 6% among male injection drug users (IDU), and 4% among men who have sex with men and inject drugs (MSM/IDU) (1). MSM/IDU might have different prevention needs from men who are either MSM or IDU, but not both. A combination of effective, scalable, and evidence-based approaches that address male-to-male sex and injection drug use behaviors might reduce HIV infections among MSM/IDU. To refine calculations of disease rates attributed to MSM and IDU (2,3) by accounting for MSM/IDU, CDC used data from 1999–2008 National Health and Nutrition Examination Survey (NHANES) to estimate the percentage and number of MSM/IDU in the general population. To further describe demographic similarities and differences of MSM/IDU identified by different surveillance systems, CDC also compared data from four HIV surveillance systems: the 2008 and 2009 National HIV Behavioral Surveillance System (NHBS), the 2011 National HIV Surveillance System (NHSS), and the 2007–2009 Medical Monitoring Project (MMP). Of males aged ≥18 years, MSM/IDU comprised an estimated 0.35% in NHANES, 7%–20% in NHBS, an estimated 4%–8% in NHSS, and 9% in MMP. Across surveillance systems, MSM/IDU accounted for 4%–12% of MSM and 11%–39% of male IDU. Risk reduction programs and interventions targeted toward male IDU populations might be more effective if they also incorporate messages about male-to-male sex.

Four national surveillance systems collect data on both male-to-male sex and injecting drug behaviors, though in differing ways (Table 1). NHANES collects data from the civilian general household population. NHBS collects data on persons at risk for HIV infection, using separate cycles for MSM and IDU. NHSS collects data on persons diagnosed with HIV infection and persons living with a diagnosis of HIV infection. MMP collects data on persons receiving medical care for HIV infection. With the exception of one data source, MSM/IDU were defined as adult males who ever had sex with a man and ever injected drugs; for the NHBS IDU cycle, MSM/IDU were defined as adult males who ever had sex with a man and injected drugs in the past 12 months (the latter being part of the NHBS IDU cycle eligibility criteria). For NHANES, data from 1999–2008 were aggregated and analyzed to obtain a robust MSM/IDU population percentage estimate, with response rates for males ranging from 69% to 72%. For other data sources, the most recent data available were analyzed. For NHSS, data were adjusted for reporting delays and missing transmission category but not for incomplete reporting. The analysis was limited to males aged ≥18 years for comparability across data sources. Differences between groups should be interpreted with caution because statistical tests were not performed.

Of the 7,011 men, representing an estimated 71,111,352 men in the adult male population (NHANES), a weighted estimate of 0.35% ever had sex with a man and ever injected drugs, and thus were classified as MSM/IDU, corresponding to 248,890 MSM/IDU. MSM comprised 5% of all men, corresponding to approximately 3,555,568 MSM. MSM/IDU comprised 7% of MSM. Similarly, IDU comprised 3% of all men, corresponding to approximately 2,133,341 IDU. MSM/IDU comprised 11% of male IDU (Table 2). Data were too few to support stratification by age or race/ethnicity.

In 2008, of 9,903 MSM interviewed for the NHBS MSM cycle, 681 (7%) were MSM/IDU (Table 2). Overall, 38% of MSM/IDU were aged 18–34 years; 65% were white, 11% black/African American, and 16% Hispanic/Latino (Table 3). In 2009, of 7,374 IDU interviewed from the NHBS IDU cycle, 1,467 (20%) were also MSM and thus classified as MSM/IDU (Table 2). Overall, 20% of MSM/IDU were aged 18–34 years; 33% were white, 36% black/African American, and 26% Hispanic/Latino (Table 3).

For 2011, NHSS data indicate there were an estimated 1,416 men diagnosed with HIV whose infections were attributed to male-to-male sex and injection drug use; these men comprised 4% of all men diagnosed with HIV infection in 2011. MSM comprised 78% of all men aged ≥18 years diagnosed with HIV infection and MSM/IDU were 4% of all MSM. IDU comprised 6% of all men diagnosed with HIV infection aged ≥18 years in 2011, and MSM/IDU comprised 38% of male IDU (Table 2). Among MSM/IDU, 51% were aged 18–34 years; 44% were white, 30% black/African American, and 21% Hispanic/Latino (Table 3). At the end of 2010, NHSS data indicated there were 49,656 adult MSM/IDU living with a diagnosis of HIV infection; these men comprised 8% of all men living with a diagnosis of HIV infection. MSM comprised 67% of all males living with a diagnosis of HIV infection, and MSM/IDU were 10% of all MSM. IDU comprised 14% of all males living with a diagnosis of HIV infection, and MSM/IDU were 35% of male IDU (Table 2). Among these MSM/IDU, 13% were aged 18–34 years; 44% were white, 34% black/African American, and 19% Hispanic/Latino (Table 3).

Among 6,635 HIV-infected men in medical care for HIV infection who participated in MMP during 2007–2009, 596 (9%) were MSM/IDU. MSM comprised 66% of all men, and MSM/IDU comprised 12% of MSM; similarly, IDU comprised 14% of all men, and MSM/IDU comprised 39% of male IDU (Table 2). Among MSM/IDU, 5% were aged 18–34 years; 46% were white, 33% black/African American, and 14% Hispanic/Latino (Table 3).

For each data source, compared with men who were not MSM/IDU, a higher proportion of MSM/IDU were white and a lower proportion were black/African American (Table 3). MSM/IDU in the NHBS MSM cycle were predominately white (65%), whereas the racial distribution among those in NHSS and MMP was more diverse: approximately 40% white, 30% black/African American, and 20% Hispanic/Latino. These data also show a large proportion (38%) of MSM/IDU in the MSM cycle of NHBS and diagnosed with HIV infection in 2011 (51%) (NHSS) were aged 18–34 years.

## Editorial Note

MSM/IDU constitute an estimated 0.35% of the general male population (248,890 men), based on data from NHANES, a general population survey of a probability sample of U.S. households. Other methods for estimating the size of HIV risk behavior populations include meta-analysis of multiple national surveys (2,3). NHANES was used because it is the largest national data source available to obtain data on both male-to-male sexual behavior and injection drug use among persons aged ≥18 years.

The findings in this report will be used in CDC’s future efforts to refine disease rates by transmission category. The findings demonstrate that, although MSM/IDU constitute only 0.35% of the general male population, they comprise 4%–12% of MSM and 11%–39% of male IDU. One study estimated the prevalence of injection drug use among MSM to be 42% (4), another estimated the prevalence of male-to-male sex among IDU to be 31% (5). In 2010, men who have sex with men, inject drugs, or do both represented 71% of persons with new HIV infections in the United States (1).

The findings in this report are subject to at least five limitations. First, using NHANES to estimate the population proportion of MSM/IDU might provide an underestimate or overestimate because institutionalized and nonhousehold-based populations are not included in the sampling strategy. Second, during 1999–2008, the response rate for sampled male participants with both an interview and a medical examination ranged from 69% in the 2001–2002 data to 72% in the 2007–2008 data, and it is unknown whether an underestimate or overestimate of the proportion of MSM/IDU would result from nonresponse. Third, MSM/IDU from the HIV surveillance systems might not be representative of all MSM/IDU in the United States (e.g., those not infected with HIV). Fourth, some participants might not have accurately reported their behaviors, which might result in underestimates of proportions of MSM/IDU. Finally, the NHSS data were adjusted statistically to account for diagnosed cases with a missing transmission category. The degree of uncertainty introduced by this imputation is unknown.

What is already known on this topic?

Men who have sex with men (MSM) and are injecting drug users (IDU) (MSM/IDU) comprise a small proportion of persons with human immunodeficiency virus (HIV) infections, but they are at increased risk for acquiring and transmitting HIV.

What is added by this report?

Using data from four national surveillance systems, the proportion of MSM/IDU was estimated to better describe the prevalence of persons engaging in both behaviors. MSM/IDU comprised an estimated 0.35% of adult males in the general household population of the United States, 7%–20% of males at high risk for HIV infection because of behaviors such as male-to-male sex or injecting drugs, 4% of males diagnosed with HIV, 8% of males living with a diagnosis of HIV infection, and 9% of males diagnosed with and in medical care for HIV infection. Across surveillance systems, MSM/IDU accounted for 4%–12% of MSM and 11%–39% of male IDU.

What are the implications for public health practice?

Risk reduction programs and interventions targeted toward male IDU populations might be more effective if they incorporate messages about male-to-male sex because 11%–39% of male IDU were also MSM in this analysis. A combination of effective, scalable, and evidence-based approaches that address male-to-male sex and injection drug use behaviors might help reduce HIV infections among MSM/IDU.

Because MSM/IDU engage in both of the HIV risk behaviors considered in this analysis, they are particularly vulnerable to infection and can transmit HIV through sexual behavior or by sharing syringes. This analysis demonstrates that the population proportion of MSM/IDU is small, but it comprises a considerable proportion of both MSM and IDU populations at risk for or infected with HIV. For persons at increased risk, such as MSM or IDU, HIV testing at least once a year is recommended (6). An integrated prevention services approach for IDU should include 1) substance abuse and mental health treatment; 2) risk reduction programs and messages, including interventions to reduce risky sexual behaviors; and 3) access to condoms and sterile injection and drug preparation equipment (7). Risk reduction programs and interventions targeted toward male IDU populations might be more effective if they incorporate messages about male-to-male sex because approximately 11%–39% of IDU also engage in male-to-male sex according to this analysis. Preexposure prophylaxis (e.g., daily doses of tenofovir disoproxil fumarate and emtricitabine) is also an appropriate prevention strategy for some high-risk IDU and MSM (8,9). The National HIV/AIDS Strategy calls for intensified HIV prevention efforts in the communities where HIV is most heavily concentrated, including blacks/African Americans, Hispanics/Latinos, gay and bisexual men, and substance abusers (10). CDC’s High Impact Prevention strategy expands efforts to prevent HIV infection using a combination of effective, scalable, and evidence-based approaches that address male-to-male sex and injection drug use behaviors that might reduce HIV infections among MSM/IDU.

## Figures and Tables

**TABLE 1:** Methods used by four surveillance systems to define survey participants as men who have sex with men (MSM) and injection drug users (IDU)

System and website	Sampling method and analysis	Data collection method	Data years	MSM/IDU	

				Definition	Defining question(s)
**National Health and Nutrition Examination Survey (NHANES) (http://www.cdc.gov/nchs/nhanes)**	Cluster-stratified, multistage probability sample of persons aged 12–59 years in U.S. households. Analysis limited to males aged 20–59 years and who gave affirmative or negative responses to questions regarding sex with a man during his lifetime and injection drug use during his lifetime. Data weighted to adjust for sampling strategy. N = 7,011 male respondents weighted to represent 71,111,352 adult men in the population.	ACASI	1999–2008	Participants who ever had sex with a man in his lifetime (>0 same sex partners) and who ever used a needle to take street drugs or used a needle to inject drugs not prescribed by a doctor.	Ever MSM (1999–2004): “In your lifetime, with how many men have you had sex?” Ever MSM (2005–2008): “In your lifetime, with how many males have you had anal or oral sex?” Ever IDU (1999–2004): “Have you ever used a needle to take street drugs?” Ever IDU (2005–2008): “Have you ever, even once, used a needle to inject a drug not prescribed by a doctor?”
**National HIV Behavioral Surveillance System (NHBS) (http://www.cdc.gov/hiv/bcsb/nhbs)**					
MSM cycle*	Time-location sampling of MSM aged ≥18 years who live in the participating MSA. Analysis sample includes male participants (n = 9,903) who gave affirmative or negative responses to questions regarding sex with a man during his lifetime and injection drug use during his lifetime. Data are unweighted.	CAPI	2008	Men who have ever had oral or anal sex with a man and who ever injected drugs.	Ever MSM: “Have you ever had oral or anal sex with a man?” Ever IDU: “Have you ever in your life shot up or injected any drugs other than those prescribed for you? By shooting up, I mean anytime you might have used drugs with a needle, either by mainlining, skin popping, or muscling.”
IDU cycle*	Respondent-driven sampling of IDUs aged ≥18 years who live in the participating MSA. Analysis sample includes male participants (n = 7,374) who gave affirmative or negative responses to questions regarding sex with a man during his lifetime and injection drug use during his lifetime. Data are unweighted.	CAPI	2009	IDU participants who reported ever having oral or anal sex with a man.	Ever MSM: “Have you ever had oral or anal sex with a man?” IDU: All eligible participants are IDU (injected in past 12 months)
**National HIV Surveillance System (NHSS) (http://www.cdc.gov/hiv/topics/surveillance)**					
HIV diagnoses	Ascertained through HIV case reporting. Analysis includes cases in persons aged ≥18 years and diagnosed in 2011 (n = 39,134). Data adjusted for reporting delays and missing transmission category but not for incomplete reporting.	Medical record review	2011	Males whose transmission category is classified as male-to-male sexual contact and injection drug use since 1977. These include men whose case report noted injecting drugs and sexual contact with other men or sexual contact with both men and women.	
Living with diagnosis of HIV	Ascertained through HIV case reporting. Analysis includes persons aged ≥18 years at time of diagnosis and living through the end of 2010 (n = 663,866). Data adjusted for reporting delays and missing transmission category but not for incomplete reporting.	Medical record review	2010	Males whose transmission category is classified as male-to-male sexual contact and injection drug use since 1977. These include men whose case report noted injecting drugs and sexual contact with other men or sexual contact with both men and women.	
Medical Monitoring Project (MMP) (http://www.cdc.gov/hiv/topics/treatment/mmp)	Three-stage probability sample (states, health-care facilities that provide HIV medical care, and patients in medical care for HIV). Analysis sample includes HIV-positive patients aged ≥18 years receiving care from HIV medical care facilities (n = 6,635). MMP data were linked to NHSS data and NHSS transmission risk was used to define MSM/IDU. Data are unweighted.	CAPI	2007–2009	Males whose transmission category is classified as male-to-male sexual contact and injection drug use since 1977. These include men whose case report noted injecting drugs and sexual contact with other men or sexual contact with both men and women.	

**Abbreviations:** ACASI = audio, computer-assisted self interview; CAPI = computer-assisted personal interview; HIV = human immunodeficiency virus; MSA = metropolitan statistical area.

* Cycle defined as data collection with a specific population.

**TABLE 2:** Percentage of males aged ≥18 years who are men who have sex with men (MSM), injection drug users (IDU), or both (MSM/IDU) — National Health and Nutrition Examination Survey (NHANES), National HIV Behavioral Surveillance System (NHBS), National HIV Surveillance System (NHSS), and Medical Monitoring Project (MMP), 1999–2011

Data source	% MSM not IDU	% IDU not MSM	% MSM/IDU	% MSM/IDU among MSM	% MSM/IDU among IDU
**NHANES 1999–2008**	4.7	3.0	0.35	6.9	10.5
(95% confidence intervals)	(4.0–5.5)	(2.4–3.5)	(0.18–0.52)	(3.6–10.1)	(6.0–15.0)
**NHBS**					
MSM cycle, 2008	93.1	—*	6.9	6.9	—*
IDU cycle, 2009	—*	80.1	19.9	—*	19.9
**NHSS**					
Diagnoses, 2011	78.1	6.0	3.6	4.4	37.5
Living with a diagnosis of HIV infection, 2010	66.8	13.8	7.5	10.1	35.1
**MMP**	66.4	14.2	8.9	11.8	38.6

**Abbreviation:** HIV = human immunodeficiency virus.

* Not applicable. The NHBS MSM cycle uses time location sampling of MSM, so there would not be men in the NHBS MSM cycle who were solely IDU. The NHBS IDU cycle uses respondent-driven sampling and only includes persons who injected drugs within the past 12 months; therefore, the NHBS IDU cycle would not include men who were solely MSM.

**TABLE 3:** Percentage of men who are both men who have sex with men (MSM) and injection drug users (IDU) (MSM/IDU), and other males, by age group and race/ethnicity — National HIV Behavioral Surveillance (NHBS), National HIV Surveillance System (NHSS), and Medical Monitoring Project (MMP), 2007–2011

	NHBS				NHSS				MMP	
			
	MSM cycle, 2008		IDU cycle, 2009		Diagnoses, 2011		Living with, 2010		In care, 2007–2009	
					
Characteristic	% MSM/IDU (n = 681)	% non-IDU (n = 9,222)	% MSM/IDU (n = 1,467)	% non-MSM (n = 5,907)	% MSM/IDU (n = 1,416)	% non-MSM/IDU (n = 37,718)	% MSM/IDU (n = 49,656)	% non-MSM/IDU (n = 614,210)	% MSM/IDU (n = 596)	% non-MSM/IDU (n = 6,089)
**Age group (yrs)**										
18–24	8.5	22.7	2.6	2.9	18.5	21.7	1.8	4.0	1.0	2.1
25–34	29.8	31.7	17.1	14.2	32.9	28.7	11.3	13.4	4.4	9.5
35–44	33.3	26.2	26.6	21.0	24.6	22.1	27.5	25.6	35.2	28.1
45–54	20.9	13.9	36.5	36.8	17.4	19.0	42.4	36.2	41.8	40.2
≥55	7.5	5.5	17.3	25.2	6.6	8.6	17.1	20.7	17.6	20.1
**Total**	**100.0**	**100.0**	**100.0**	**100.0**	**100.0**	**100.0**	**100.0**	**100.0**	**100.0**	**100.0**
**Race/Ethnicity**										
White	65.3	42.1	32.9	24.1	43.8	30.2	43.7	37.9	46.1	40.0
Black	11.3	24.8	35.5	49.5	30.3	41.9	33.8	37.3	32.9	35.1
Hispanic	16.0	24.2	25.7	22.8	20.9	23.5	18.6	21.5	14.3	18.9
American Indian/Alaska Native	1.2	0.5	1.0	0.8	0.9	0.4	0.8	0.3	1.5	0.6
Asian	0.4	2.3	0.3	0.3	1.7	2.1	0.7	1.2	0.0	0.8
Native Hawaiian or other Pacific Islander	0.2	0.7	0.3	0.2	0.0	0.2	0.1	0.1	0.7	0.3
Multirace	4.3	3.6	4.4	2.5	2.4	1.7	2.2	1.5	3.2	3.0
Other/Unknown	1.3	1.7	0.0	0.0	0.0	0.0	0.1	0.1	1.3	1.2
**Total**	**100.0**	**100.0**	**100.0**	**100.0**	**100.0**	**100.0**	**100.0**	**100.0**	**100.0**	**100.0**
